# Three variations of the laryngeal nerve in the same patient: a case report

**DOI:** 10.1186/1752-1947-5-266

**Published:** 2011-07-01

**Authors:** Emin Gurleyik

**Affiliations:** 1 Department of Surgery, Duzce University, Medical Faculty, Duzce, Turkey

## Abstract

**Introduction:**

A non-recurrent course is a rare anatomic variation of the inferior laryngeal nerve (ILN). Bilateral extra-laryngeal bifurcation of the ILN seldom occurs before its laryngeal entry. Anastomosis between the ILN and cervical sympathetic chain is another rare anatomic feature. The prevalence of extra-laryngeal branching of the non-recurrent nerve is unknown. We present an example of triple anatomic variations of ILNs in the same patient, and also two anatomic variations in the same nerve.

**Case presentation:**

A 56-year-old Caucasian man with a large toxic multi-nodular goiter was surgically treated with total thyroidectomy. Both his right and left ILNs were identified, fully exposed and preserved along their cervical courses. We discovered many variations during bilateral exploration of the two ILNs. His right ILN was non-recurrent. This non-recurrent ILN showed a terminal division before laryngeal entry. The left nerve had a usual course as a recurrent laryngeal nerve (RLN) at his tracheaesophageal groove. We also discovered bifurcation of his RLN beginning at a neurovascular (RLN and inferior thyroid artery) crossing point. Anterior and posterior branches of both nerves entered his larynx separately. The sympathetic inferior laryngeal anastomotic branch (SILAB) between the posterior branch of his left ILN and the cervical sympathetic chain was established in the distal part of the nerve before laryngeal entry.

**Conclusion:**

A non-recurrent nerve and extra-laryngeal branching of the ILN are two different variations. The coincidence of a right non-recurrent ILN and bilateral bifurcation of both nerves is a very interesting feature. SILAB is a rare additional finding as a third anatomic variation in the same patient. Extra-laryngeal terminal division of a non-recurrent ILN is an extremely unusual anatomic finding. Two anatomic variations have occurred in the same nerve, like "the variation of the variation".

## Introduction

The inferior laryngeal nerve (ILN) is the most important structure in thyroid operations. Anatomic variations of a recurrent laryngeal nerve (RLN) may threaten the safety of thyroid surgery and so a complete knowledge of RLN anatomy, including all of its variations, must be mandatory for thyroid surgeons. Extra-laryngeal branching of the RLN as a terminal division is a common anatomic variation macroscopically discovered at surgical exploration. Terminal division of both right and left nerves seldom occurs bilaterally. Larger branches of the nerve bifurcation may affect laryngeal function. Non-recurrent ILN is a rare and important anatomic variation, threatening the safety of thyroid surgery [1-7]. The association of a non-recurrent nerve and extra-laryngeal branching is an extremely rare occurrence. An anastomosis between an ILN and the cervical sympathetic chain is another rare anatomic variation, known as sympathetic inferior laryngeal anastomotic branch (SILAB) [6,8].

In this case report, three variations of ILN, with two variations in one ILN, are presented in the same patient with a large goiter surgically treated with total thyroidectomy.

## Case Report

A 56-year-old Caucasian male patient with a goiter, situated in an endemic goiter region, had a 40-year history of thyroid gland enlargement. He presented to the department of surgery with a large goiter and symptoms of hyperthyroidism.

A physical examination, blood chemistry for thyroid-stimulating hormone and free thyroxin, ultrasound imaging and thyroid nuclear scanning established the diagnosis of toxic multi-nodular goiter. Thyroid hyperfunction was normalized with anti-thyroid medication.

Our patient was treated with a total thyroidectomy. The weight of the fresh thyroid specimen was 404 g. The inferior thyroid arteries were identified, isolated, and a loop of silk suture was placed around the artery for traction. His ILN was identified below the artery and fully isolated on the left side after freeing and medially mobilizing the thyroid gland. His right ILN was not found at the usual position.

**Figure 1:**
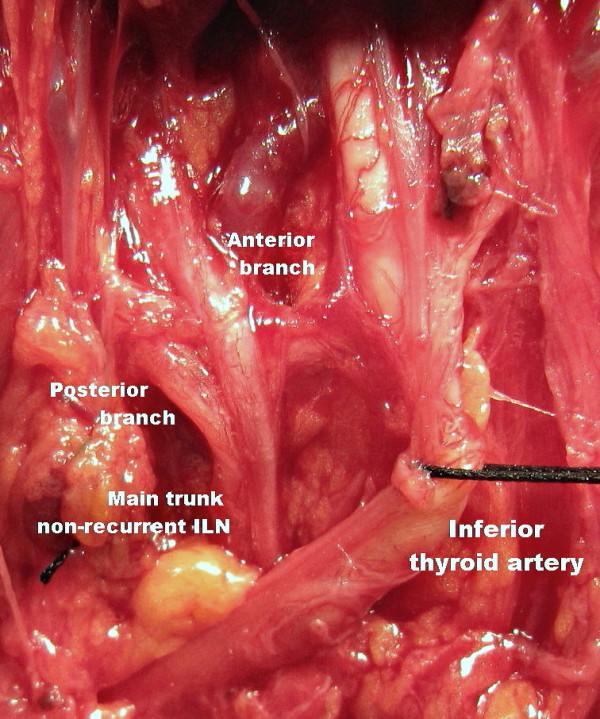
**Right non-recurrent ILN with extra-laryngeal bifurcation (magnified and edited operative photograph)**.

An exploration was made for his right ILN at the anticipated crossing point of the nerve and the artery. His right RLN was found not to follow the usual course of the nerve in the tracheaesophageal groove. The dissection was advanced in an upward direction through the Berry ligament. A non-recurrent laryngeal nerve was identified and exposed near the Berry ligament. The non-recurrent nerve had a course parallel to that of his inferior thyroid artery. The nerve had extra-laryngeal terminal division, as anterior and posterior branches (Figure 1). The laryngeal entry point of the non-recurrent nerve was the same as the RLN. The two branches entered the larynx separately.

**Figure 2:**
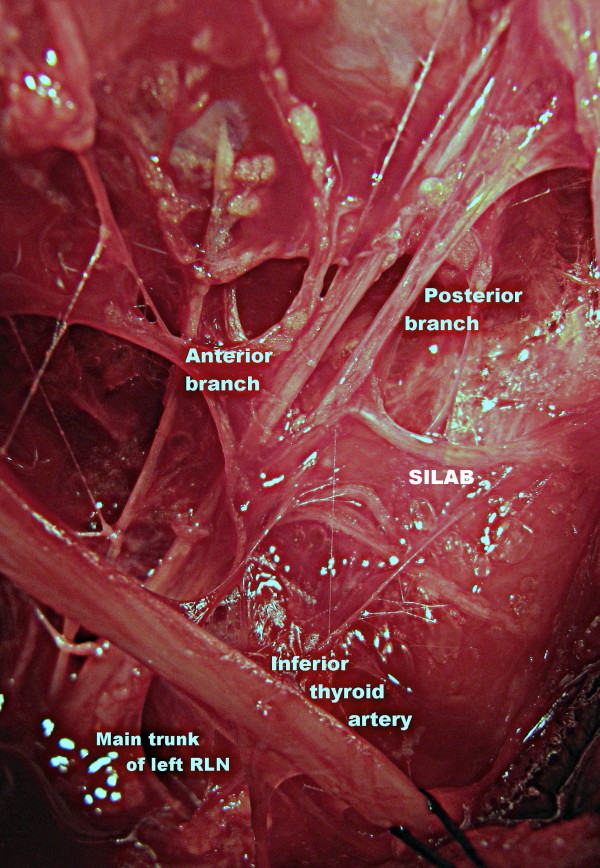
**Left RLN bifurcated at artery and nerve crossing point**. A horizontal nerve anastomosis on the posterior branch of the RLN known as SILAB (magnified and edited operative photograph).

**Figure 3:**
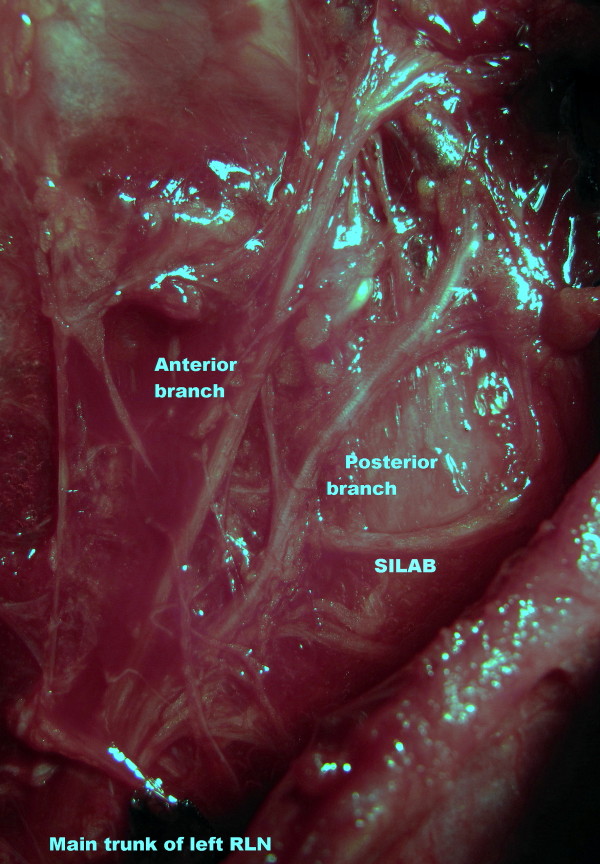
**Left RLN with extra-laryngeal terminal division**. Each branch enters the larynx separately. Horizontal nerve anastomosis on the posterior branch of the RLN known as SILAB (magnified and edited operative photograph).

After isolation of his left inferior thyroid artery, his left RLN was explored at the anticipated neurovascular crossing point. The main trunk of his left RLN was first identified caudal to the artery. His left RLN was dissected in an upward direction to the laryngeal entry point, for full exposure of the nerve. During surgical dissection, the left RLN was found to have extra-laryngeal branching at the neurovascular crossing point where the nerve medially passes the inferior thyroid artery. Anterior and posterior branches of his left RLN entered the larynx separately. During routine exploration, we incidentally found an anastomotic nerve branch with a horizontal course on the posterior branch of the left RLN before laryngeal entry (Figures 2 and Figure 3).

## Discussion

The safety of thyroid operations mainly depends on the surgical anatomy of the ILN. The nerve has many variations on its cervical course and full knowledge of RLN anatomy, including all variations, is obligatory for a thyroid surgeon. Variations of the ILN, such as rare non-recurrent ILN, threaten the safety of thyroid operations [5-7]. SILAB is another rare anatomic variation [6,8]. On the other hand, extra-laryngeal terminal division of RLN is a common variation macroscopically discovered during thyroid operations [2,3,9,10]. Our patient is a rare example of triple ILN variations; co-existence of bilateral extra-laryngeal bifurcation of ILNs, right non-recurrent nerve and left SILAB. Synchronicity of three variations of the ILN in one patient is an extremely rare coincidence.

The prevalence of non-recurrent nerve has been reported below 1% [5-7] and increases the risk of ILN injury. The laryngeal entry point for a non-recurrent ILN is the same as for a recurrent nerve. If the RLN is not found in its normal course during surgical exploration, the possibility of a non-recurrent nerve must be taken into account. Complete exposure of the RLN is mandatory to avoid nerve injury; especially near the Berry ligament where the nerve is more superficial and vulnerable during total thyroidectomy. The main anatomical feature of our patient was the extra-laryngeal terminal division of a right non-recurrent nerve. The prevalence of bifurcated non-recurrent ILN is unknown; two variations have occurred in the same nerve. This is a "variation of the variation". This double variation in the same nerve is extremely unusual.

Extra-laryngeal branching of RLN is a common variation reported in up to 65% of surgical and autopsy series [1-3,10]. Macroscopically discovered terminal division of the nerve during thyroid operations has a prevalence of 18-36% [2,3,9,10]. Bilateral bifurcation of both RLNs is relatively rare with a prevalence of 3-9% [9,10]. This bifurcation, in larger anterior and posterior branches, may have significant functions. The anterior branch has motor fibers, whilst the posterior branch is mainly sensitive [4,9]. In some cases, the posterior branch also has motor fibers, and may affect laryngeal function. It may innervate posterior cricoarytenoid (abductor function) and interarytenoid muscles [4]. Extra-laryngeal terminal division of the ILN must be identified, exposed and preserved for motor function. The more superficial anterior branch, the principal motor division of the nerve, is under a greater risk of injury during Berry ligament dissection.

The SILAB is an anastomotic branch from the middle or superior cervical sympathetic ganglion to the RLN. The prevalence of SILAB has been reported as 0.74-1.5%. A large SILAB, with its horizontal course, may be mistaken for a non-recurrent nerve [6,8]. This branch brings sympathetic fibers to the ILN [4,6,8]. A left SILAB, which was localized in last 2-3 cm of the RLN, was discovered as a third variation of the ILN in our patient.

## Conclusion

The co-existence of three variations of the ILN, a right non-recurrent nerve, bilateral extra-laryngeal bifurcation of the ILN and left SILAB, is an extremely unusual coincidence in one patient. The coincidence of a right non-recurrent nerve and bilateral bifurcation of both right and left nerves in the same patient is a very interesting feature. SILAB appeared as additional third variation of RLN in our patient. Extra-laryngeal bifurcation of the non-recurrent ILN is an extremely unusual anatomic finding. Association of two anatomic variations has occurred in the same nerve, presenting "the variation of the variation".

## Abbreviations

ILN: Inferior laryngeal nerve; RLN: Recurrent laryngeal nerve; SILAB: Sympathetic inferior laryngeal anastomotic branch.

## Consent

Written informed consent was obtained from the patient for publication of this case report and any accompanying images. A copy of the written consent is available for review by the Editor-in-Chief of this journal.

## Competing interests

The authors declare that they have no competing interests.
